# Microbial Community Profiling of Concrete

**DOI:** 10.3390/microorganisms14010131

**Published:** 2026-01-07

**Authors:** Caroline Danner, Julien Charest, Carlijn Borghuis, Philipp Aschenbrenner, Jakob Lederer, Robert L. Mach, Astrid R. Mach-Aigner

**Affiliations:** 1 Institute of Chemical, Environmental and Bioscience Engineering, Technische Universität Wien, Gumpendorfer Strasse 1a, 1060 Wien, Austria; caroline.danner@tuwien.ac.at (C.D.); carlijn.borghuis@gmail.com (C.B.); jakob.lederer@tuwien.ac.at (J.L.); robert.mach@tuwien.ac.at (R.L.M.); astrid.mach-aigner@tuwien.ac.at (A.R.M.-A.); 2 Institute of Water Quality and Resource Management, Technische Universität Wien, Karlsplatz 13, 1040 Wien, Austria; philipp.aschenbrenner@tuwien.ac.at

**Keywords:** microbial communities, concrete, concrete recycling, concreate healing

## Abstract

Concrete is the most widely used construction material worldwide, yet its production and disposal pose significant environmental challenges due to high carbon emissions and limited recyclability. While microbial colonization of concrete is often associated with structural deterioration, recent research has highlighted the potential of microorganisms to contribute positively to concrete recycling and self-healing. In this study, we investigated the bacterial and fungal communities inhabiting urban concrete samples using amplicon-based taxonomic profiling targeting the 16S rRNA gene and internal transcribed spacer (ITS) region. Our analyses revealed a diverse assemblage of microbial taxa capable of surviving the extreme physicochemical conditions of concrete. Several taxa were associated with known metabolic functions relevant to concrete degradation, such as acid and sulphate production, as well as biomineralization processes that may support crack repair and surface sealing. These findings suggest that concrete-associated microbiomes may serve as a reservoir of biological functions with potential applications in sustainable construction, including targeted biodegradation for recycling and biogenic mineral formation for structural healing. This work provides a foundation for developing microbial solutions to reduce the environmental footprint of concrete infrastructure.

## 1. Introduction

Concrete, a ubiquitous and durable composite material composed of cement (primarily tricalcium silicate (3CaO·SiO_2_), dicalcium silicate (2CaO·SiO_2_), tricalcium aluminate (3CaO·Al_2_O_3_), and tetra-calcium aluminoferrite (4CaO·Al_2_O_3_Fe_2_O_3_), fine and coarse aggregates, water, and optionally other additives, has played a pivotal role in shaping modern infrastructures due to its versatility, strength, and moldability [1]. Despite its engineering advantages, the environmental impact of concrete production is significant: the production of cement alone accounts for a considerable share of global CO_2_ emissions and requires the extraction of finite natural resources [2]. These concerns have catalyzed efforts to develop sustainable alternatives, such as the use of low-carbon cementitious materials and recycled aggregates; however, recycling concrete remains a technical and logistical challenge. Demolished structures are typically crushed into smaller particles for reuse as aggregate in low-grade applications, often referred to as downcycling [3,4]. Moreover, contaminants such as embedded steel, wood, and chemical additives complicate processing, reducing the efficiency and quality of recycled concrete [5,6]. New strategies are needed to improve the recyclability and functional reuse of concrete materials. Among the emerging approaches is the use of biological agents to facilitate selective degradation or mineral recovery, offering a potentially more targeted and environmentally friendly solution.

Biological interactions with concrete have traditionally been viewed through the lens of deterioration. Concrete, although largely inert, can support microbial colonization under favorable environmental conditions, including low surface pH, elevated relative humidity (i.e., between 60–98%), long cycles of humidification and drying, freezing and defrosting, high CO_2_ concentration (e.g., carbonation in urban atmospheres), high salt concentration or high concentration of sulfates and small amounts of acids [7]. The biodeterioration of concrete can occur through various mechanisms, such as production of organic acids (e.g., acetic, lactic, and butyric acid), which can lower the pH levels and initiate the dissolution of minerals within the concrete, contributing to the erosion of the exposed concrete surface, reducing the protective cover depth, increasing concrete porosity and increasing the transport of degrading agents in the structure [8]. For example, sulphur-oxidizing bacteria oxidize H_2_S to sulphuric acid, leading to accelerated corrosion in concrete-applications such as sewer systems and barn floors [9]. Other microbes secrete enzymes that degrade organic components in the cement paste, exacerbating porosity and structural weakening [10]. Despite this negative impact, the metabolic versatility of these organisms also offers a promising avenue for biotechnological exploitation. Recent interest has shifted toward harnessing microbes not just as agents of degradation, but also as contributors to concrete repair and sustainability. Certain microbial taxa can induce calcium carbonate precipitation, a phenomenon that can be exploited for crack healing and surface sealing [11]. Such “self-healing concrete” technologies rely on microbial metabolism to form new mineral phases that restore structural integrity. However, a detailed understanding of the microbial communities inhabiting concrete and their functional capabilities remains limited.

In this study, we employed amplicon-based taxonomic profiling to investigate the bacterial (16S rRNA gene) and fungal (ITS region) communities associated with concrete samples collected from diverse urban environments. Our goal was to identify resident taxa capable of colonizing urban concrete, therefore providing a shortlist of organisms that may be leveraged as starting points for future biotechnological applications with minimal engineering. By linking community composition with known or putative microbial activities relevant to cement degradation or mineral precipitation, we aim to provide a foundation for the development of sustainable strategies for controlled concrete recycling and biomineralization-based self-healing.

## 2. Materials and Methods

### 2.1. Sample Acquisition

To characterize microbial communities associated with concrete in urban environments, seven concrete samples were provided by the City of Vienna (Stadt Wien, MA 39, Austria), collected from various locations in and around the City of Vienna, Austria (Table 1). Samples 1 and 2 had been previously processed by the construction material testing laboratory of the City of Vienna (Stadt Wien, MA 39), including heat-drying, and were stored under dry conditions for more than three months before analysis. Sample 3 was collected from a relatively humid underground parking facility and exhibited early signs of surface deterioration. Samples 4 and 5 were obtained from two exterior demolition sites and were in comparatively good condition. Samples 6 and 7 were collected from two neighboring demolition sites. All samples were handled using sterile tools at the time of collection and were stored at −20 °C until DNA extraction.

### 2.2. Metagenomic DNA Extraction

Metagenomic DNA was extracted from seven concrete samples using the DNeasy PowerMax Soil Kit [QIAGEN, Hilden, Germany, Cat. No. 12988-10], which is optimized for isolating microbial DNA from large quantities of material with low microbial biomass. For each sample, a small portion of the original concrete block was aseptically crushed in a sterile environment, and 10 g of the resulting pulverized material was processed according to the manufacturer’s protocol. Following extraction, the total DNA was concentrated 100-fold by precipitation with 5 M sodium acetate and 0.7 volumes of isopropanol. The precipitated DNA was then pelleted by centrifugation, washed with 70% ethanol, air-dried, and resuspended in nuclease-free water.

### 2.3. Library Preparation and Sequencing

To characterize bacterial and fungal communities, unsaturated PCR amplification of 16S and ITS rDNA fragments was performed using the Q5 High-Fidelity DNA Polymerase [New England Biolabs, Ipswich, MA, USA, Cat. No. M0491], which provides ultra-low amplification error rates. For bacteria, the V3–V4 region of the 16S rRNA gene was targeted using primers 341F (5′-CCTAYGGGRBGCASCAG-3′) and 806R (5′-GGACTACNNGGGTATCTAAT-3′) [12]. For fungi, the ITS1 region was amplified using primers ITS5-1737F (5′-GGAAGTAAAAGTCGTAACAAGG-3′) and ITS2-2043R (5′-GCTGCGTTCTTCATCGATGC-3′) [12]. PCRs were performed in 50 µL reactions using 1 µL of template DNA (~0.5 ng total DNA per reaction). Thermal cycling conditions were 98 °C for 30 s, followed by 25 cycles of 98 °C for 8 s, 57 °C for 20 s, and 72 °C for 15 s, with a final extension at 72 °C for 2 min. For each sample, PCRs were performed in four replicate 50 µL reactions, which were pooled prior to purification and library preparation. All PCR reactions included a no-template control using nuclease-free water and reagents from the DNeasy PowerMax Soil Kit and DNA precipitation steps, which yielded no detectable amplification, confirming the absence of contamination. Amplicons were purified using the GeneJET Gel Extraction Kit [Thermo Fisher Scientific, Waltham, MA, USA, Cat. No. K0691] prior to library preparation. Libraries were constructed using the NEBNext Ultra II DNA Library Prep Kit for Illumina [New England Biolabs, Cat. No. E7645] following the manufacturer’s instructions. Indexed libraries were multiplexed and sequenced in-house on an Illumina MiSeq system using a MiSeq v2 300-cycle flow cell [Illumina, San Diego, CA, USA, Cat. No. MS-102-2002], generating single-end 300 bp reads (Table 2).

### 2.4. Microbial Community Composition Analysis

Following sequencing, the quality of raw 16S and ITS amplicon reads was assessed using FastQC (v0.11.9) [13]. Sequencing adapters and low-quality reads were trimmed and removed using Cutadapt (v5.2) [14]. Amplicon sequence data were processed using the DADA2 pipeline (v1.26.0) [15] in R (v4.5.0), which performs quality filtering, dereplication, sample-specific error modelling, inference of amplicon sequence variants (ASVs), and chimera removal (Table 2). Taxonomic assignment was conducted using the SILVA reference database (release 138.2) [16] for bacterial 16S rRNA sequences and the UNITE database (version 10) [17] for fungal ITS sequences, and structured using the phyloseq package (v1.52.0) [18]. Prior to diversity analyses, ASV tables were transformed to relative abundances to account for unequal sequencing depth. Alpha-diversity metrics were computed in phyloseq, and beta diversity was assessed using Bray–Curtis dissimilarities and NMDS ordination.

### 2.5. Concrete Sample Preparation and Analysis of Physicochemical Properties

Concrete samples were crushed to 4 mm grains using a BB200 heavy-metal free Jaw crusher [Retsch, Haan, Germany, Cat. No. 20.059.0001] and then further milled to a grain size of less than <100 µm with a vibrating-disc mill [Fritsch, Idar-Oberstein, Germany, Cat. No. 09.5000.00]. The pH of concrete leachate was measured for both grain sizes. Crushed or milled concrete was properly mixed, triplicate samples of 5 g were taken using the quartering sampling method [19], and dissolved in 50 mL of tap water. Samples were kept on a wave platform shaker set to 100 rpm, and pH was recorded at 30 min, 90 min, and 72 h. The elemental composition was analyzed with the NitonTM XL3t X-Ray Fluorescence (XRF) Analyzer [Thermo Scientific, Waltham, MA, USA, Cat. No. 10131166] using the milled concrete samples.

### 2.6. Multivariate Analysis of Microbial Community Composition and Concrete Chemistry

Bacterial and fungal communities were analyzed together to explore associations between microbial composition and the chemical properties of concrete. Amplicon sequence variant (ASV) count tables and associated sample metadata were imported into R (v4.5.0) and structured using the phyloseq package (v1.52.0) [18]. Ordination was performed using non-metric multidimensional scaling (NMDS) implemented with the vegan package (v2.7-1) to visualize variation in community structure [20]. Family-level counts were converted to relative abundances using decostand(method = “total”), and Bray–Curtis dissimilarities were calculated prior to NMDS using metaMDS(). Environmental variables describing elemental composition were standardized (z-transformed) and fitted to the ordination using the envfit() function. Because the number of possible permutations exceeded the requested number, envfit() performed a free permutation test by generating the full set of permutations available for the dataset (5039 total). Vector direction and length represent the orientation and strength (r^2^) of correlations between individual variables and the NMDS configuration.

## 3. Results

### 3.1. Bacterial Community Composition

**Figure 1:**
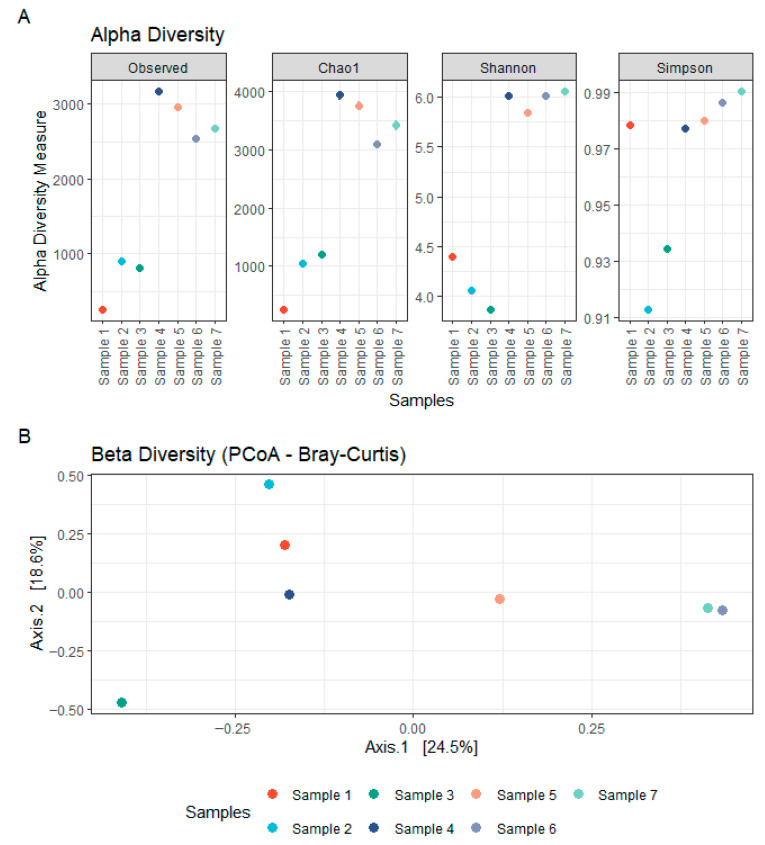
**Bacterial diversity within concrete samples.** (**A**) Alpha diversity of bacterial communities in concrete, assessed using Observed, Chao1, Shannon, and Simpson indices based on 16S rDNA sequencing. (**B**) Beta diversity of bacterial communities in concrete, visualized using Principal Coordinates Analysis (PCoA) based on Bray–Curtis distances from 16S rDNA sequencing data.

**Figure 2:**
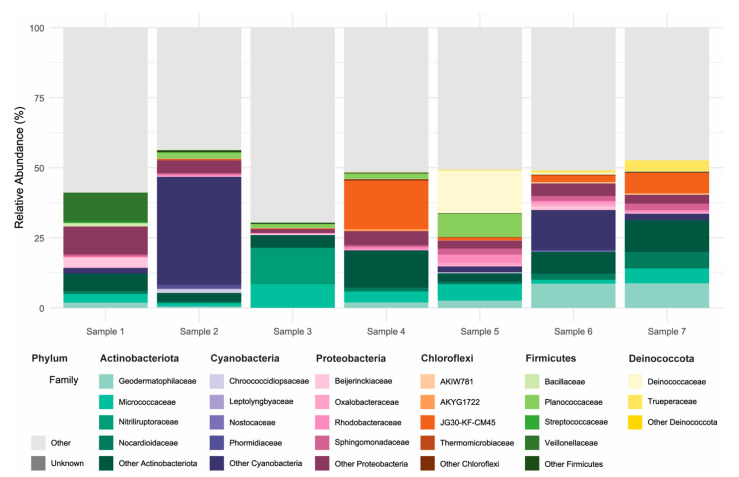
**Taxonomic composition of bacterial communities in concrete samples.** Relative abundance (%) of bacterial ASVs based on 16S rDNA sequencing, shown at the family level for the six most abundant phyla: Actinobacteriota, Cyanobacteria, Proteobacteria, Chloroflexi, Firmicutes, and Deinococcota. For each phylum, the four most abundant families are displayed individually; remaining families within the same phylum are grouped as “Other [Phylum]”. Taxa from less abundant phyla are grouped under “Other”, and ASVs not assigned to a known phylum are labeled as “Unknown”.

The bacterial communities present in the concrete samples were highly diverse and exhibited marked variation across sites. Alpha diversity estimates based on observed richness and the Chao1 index were higher in samples 4–7 than in samples 1–3, indicating a greater species richness in these communities (Figure 1). Shannon and Simpson indices showed a similar trend, reflecting the richness and evenness of the community compositions, suggesting a more diverse and balanced distribution of taxa in samples 4–7. Samples 1–2 were heat-dried and stored prior to processing, whereas samples 3–7 were processed fresh. Despite lower alpha-diversity in samples 1–2 (and partially sample 3) relative to samples 4–7, taxonomic profiles remained broadly overlapping across samples (Supplementary Table S1), including several genera detected in all samples; the potential influence of heat-drying and storage is discussed below. Taxonomic profiling of the 16S rRNA gene sequences revealed a broad range of bacterial phyla, with the most prominent lineages including Actinobacteriota, Cyanobacteria, Proteobacteria, Chloroflexi, Firmicutes, and Deinococcota (Figure 2). Actinobacteriota comprised between 5.4% and 31.0% of the total classified reads and were consistently among the dominant phyla across all samples (Supplementary Table S1). Within this group, the orders Micrococcales (2.0–11.8%) and Frankiales (0.1–14.4%) were especially abundant. These were primarily represented by the families Micrococcaceae (1.1–8.3%), Microbacteriaceae (0.006–3.7%), Intrasporangiaceae (0.06–4.6%), and Geodermatophilaceae (0.005–8.9%). Cyanobacteria were highly variable across samples, accounting for as little as 0.005% and up to 40.9% of the total reads. Most cyanobacterial reads were classified as Chloroplasts (0.003–38.0%), with smaller fractions assigned to Cyanobacteriales (up to 2.9%). Proteobacteria, another major phylum observed in all samples, ranged from 2.3% to 14.6% of the bacterial community. The most represented classes were Alphaproteobacteria (0.9–7.1%) and Gammaproteobacteria (0.3–2.8%). Other phyla, such as Chloroflexi (0.2–18.7%), Firmicutes (0.01–12.1%), and Deinococcota (0.001–15.4%), were also consistently detected, although their relative abundance varied widely between samples. Additional minor taxa included Patescibacteria, Bacteroidota, Acidobacteriota, Fusobacteriota, Verrucomicrobiota, Gemmatimonadota, Crenarchaeota, Planctomycetota, Sumerlaeota, Myxococcota, and Bdellovibrionota, each contributing less than 5% of the community in most samples. Remarkably, a large proportion of the detected amplicon sequence variants (42.2–68.4%) could not be confidently assigned to known taxonomic groups, suggesting the presence of uncharacterized or poorly represented taxa in reference databases. These findings underscore the phylogenetic complexity of microbial communities inhabiting concrete structures and point toward a substantial fraction of potentially novel or uncultured bacteria within this environment.

### 3.2. Fungal Community Composition

**Figure 3:**
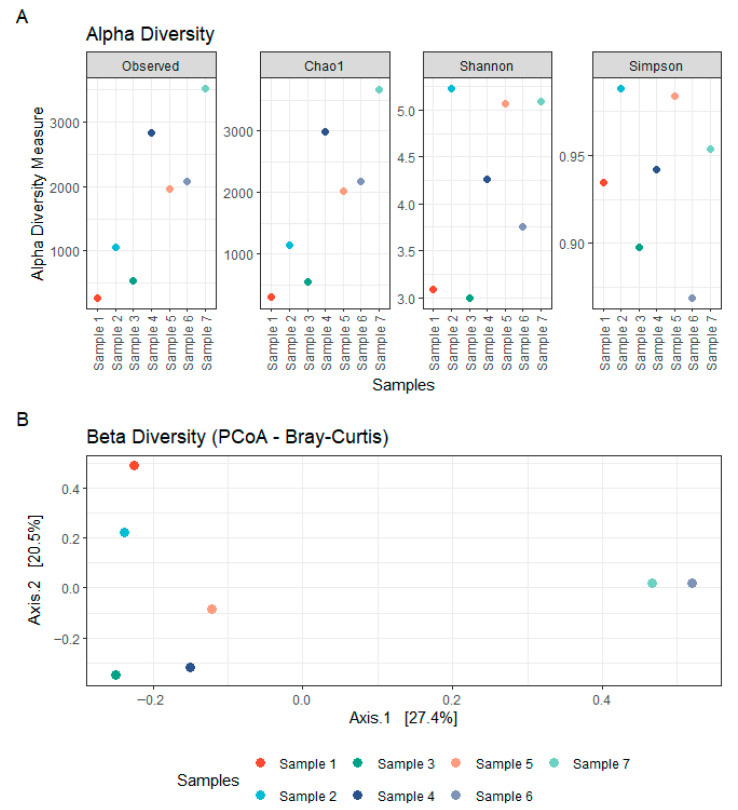
**Fungal diversity within concrete samples.** (**A**) Alpha diversity of fungal communities in concrete, assessed using Observed, Chao1, Shannon, and Simpson indices based on rDNA ITS sequencing. (**B**) Beta diversity of fungal communities in concrete, visualized using Principal Coordinates Analysis (PCoA) based on Bray–Curtis distances from rDNA ITS sequencing data.

**Figure 4:**
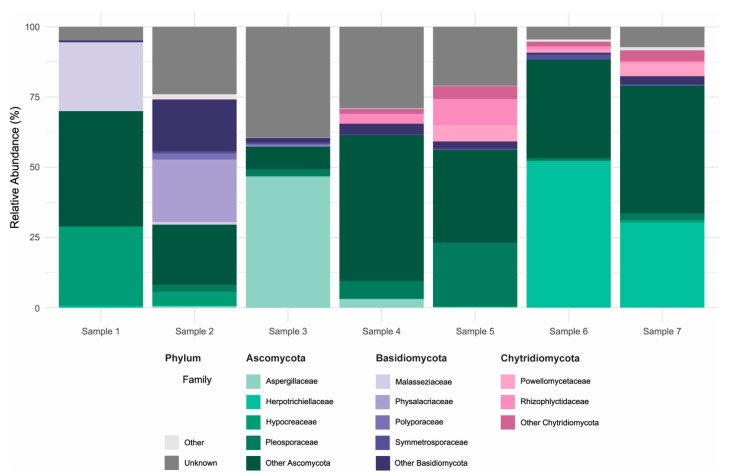
**Taxonomic composition of fungal communities in concrete samples.** Relative abundance (%) of fungal ASVs based on rDNA ITS sequencing, shown at the family level for the three most abundant phyla: Ascomycota, Basidiomycota and Chytridiomycota. For each phylum, the four most abundant families are displayed individually; remaining families within the same phylum are grouped as “Other [Phylum]”. Taxa from less abundant phyla are grouped under “Other”, and ASVs not assigned to a known phylum are labeled as “Unknown”.

The fungal communities associated with the concrete samples exhibited high variability in both composition and diversity across sites. Alpha diversity estimates, based on Observed richness, as well as Chao1, Shannon and Simpson indices, revealed substantial fluctuations among samples, indicating marked differences in fungal richness and evenness (Figure 3). Samples 1–2 were heat-dried and stored prior to processing, wheras samples 3–7 were processed fresh. Despite lower alpha-diversity in samples 1–2, and partly sample 3), ITS taxonomic profiles showed broad overlap across samples (Supplementary Table S2), including seven genera detected in all samples; potential effects of heat-drying and storage are addressed in the discussion. Taxonomic classification of the ITS sequences identified a broad range of fungal phyla, with the dominant groups being Ascomycota (29.7–88.3% of classified reads) and Basidiomycota (2.4–44.4%), followed by lower-abundance lineages including Chytridiomycota (0.001–19.8%), Rozellomycota (0.000–1.5%), Blastocladiomycota (up to 0.7%), and Monoblepharomycota (up to 0.4%) (Figure 4; Supplementary Table S2). Other phyla collectively contributed less than 0.05% of the classified reads. Notably, a substantial fraction of ASVs (4.5–39.5%) could not be confidently assigned to known taxa, suggesting the presence of potentially novel or poorly characterized fungal lineages. Within the Ascomycota, the class Sordariomycetes was particularly well represented, accounting for 4.0–40.2% of total reads. This was largely due to high relative abundances of the genera *Trichoderma* (0.015–28.3%, family Hypocreaceae) and *Fusarium* (0.03–11.9%, family Nectriaceae). Eurotiomycetes also constituted a significant portion of the community in several samples (0.7–57.7%), primarily driven by members of the genus *Exophiala* (0.008–52.2%, family Herpotrichiellaceae), which emerged as the most abundant genus overall. Additional contributions within this class came from *Penicillium* (0.000–28.7%) and *Aspergillus* (0.000–18.0%), both belonging to the Aspergillaceae family. Among the Basidiomycota, the families Malasseziaceae (0.001–24.6%) and Physalacriaceae (up to 22.1%) were the most frequently detected. In particular, the presence of *Malassezia*, a genus typically associated with skin and other moist environments, highlights the ability of some members of this phylum to colonize and persist in unconventional substrates such as concrete. Other noteworthy phyla included Chytridiomycota, in which the orders Spizellomycetales (up to 8.3%) and Rhizophlyctidales (up to 9.4%) were most prominent. Although often considered aquatic or soil-associated, their detection in concrete suggests the presence of microhabitats that may support their growth and persistence. Together, these findings highlight the remarkable phylogenetic breadth of fungi colonizing concrete structures and point to both cosmopolitan taxa and site-specific specialists potentially adapted to the harsh physicochemical conditions of this niche.

### 3.3. Concrete Physicochemical Composition and Its Relation to Microbial Communities

**Figure 5:**
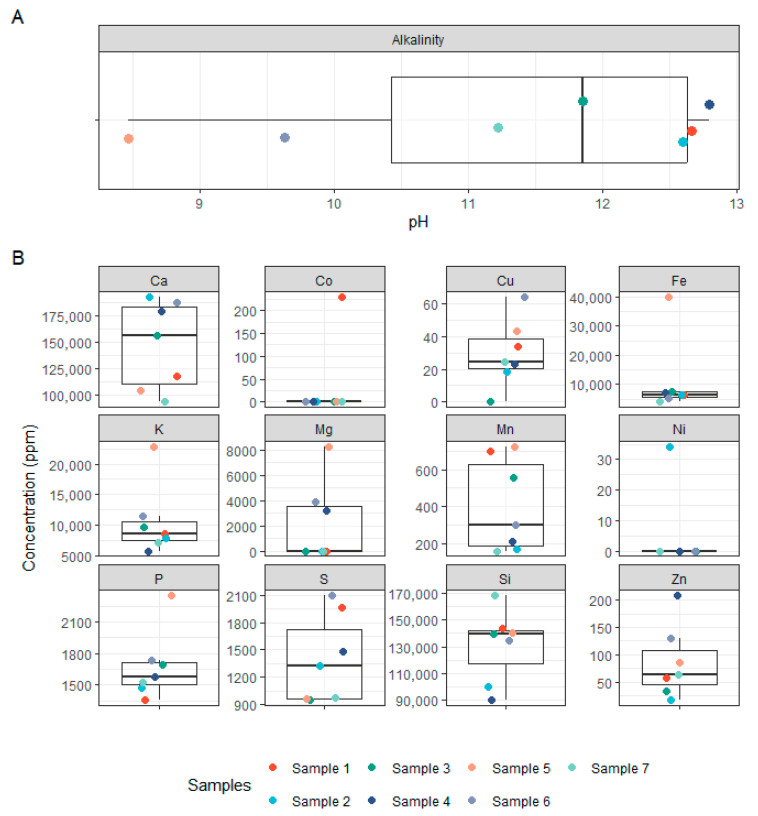
**Physicochemical properties of concrete samples.** (**A**) Boxplot showing the pH values of the concrete samples. (**B**) Boxplots of elemental concentrations (ppm) for selected major and trace elements measured in concrete samples, including Ca, Si, Fe, K, P, S, Mg, Cu, Zn, Mn, Co, and Ni.

The physicochemical analysis of the seven concrete samples revealed substantial variability across samples in both pH and elemental composition. Most samples exhibited highly alkaline pH values, ranging from 11.73 to 12.80, consistent with the expected chemical environment of concrete (Figure 5). However, two samples (5 and 6) showed markedly lower pH values of 8.47 and 9.63, respectively, suggesting potential aging or environmental exposure effects. Calcium (Ca), a major component of cementitious materials, was present in high concentrations across all samples (93,000 to 192,000 ppm), with no clear pattern across pH gradients (Figure 5). Silicon (Si) was similarly abundant (90,000 to 168,000 ppm), aligning with the mineral matrix of concrete. Other key elements such as potassium (K), iron (Fe), phosphorus (P), and sulphur (S) also showed marked variation across samples. Notably, Fe ranged from 4000 to 40,000 ppm, while K concentrations peaked at 23,000 ppm in sample 5. Magnesium (Mg) was below the limit of detection (LOD) in most samples except 4–6, where it reached up to 8000 ppm. Transition metals such as copper (Cu), manganese (Mn), zinc (Zn), cobalt (Co), and nickel (Ni) were present in variable and often trace amounts. Cobalt was detected only in sample 1 (230 ppm), and nickel only in sample 2 (34 ppm), while other samples were below LOD. Zinc concentrations were highest in sample 4 (208 ppm), with the other samples ranging from ~17 to 130 ppm. Supplementary Figure S1 and Supplementary Table S3 provide a complete overview of all measured elements. Pairwise correlations between element abundances are displayed in the triangular correlation matrix in Supplementary Figure S2. Despite the observed chemical heterogeneity, no significant correlation was found between the concrete composition and the structure of bacterial or fungal communities, as assessed using multivariate statistical approaches (NMDS stress = 7.19 × 10^−5^; see Supplementary Figure S3 and Supplementary Table S4). This is likely due to the limited number of samples and the high variability in physicochemical profiles. Instead, beta-diversity analyses (Figure 1B and Figure 3B) indicated that microbial community similarity more closely reflected the geographical proximity of the sampling sites, suggesting that environmental factors and local dispersal patterns, such as spore availability, played a stronger role in shaping microbial communities than the concrete’s chemical environment.

## 4. Discussion

We used 16S rRNA gene and ITS amplicon sequencing to profile bacterial and fungal communities across seven urban concrete samples, with the aim of identifying resident taxa already capable of colonizing and persisting in concrete, and thus potential candidates for applications that leverage intrinsic adaptation and require minimal engineering, such as biogenic crack sealing via biomineralization or controlled microbially mediated degradation for recycling. Two samples (Samples 1–2) were heat-dried and stored prior to acquisition, whereas Samples 3–7 were processed fresh. DNA yields from the heat-dried samples were comparable to those from fresh concrete, but alpha-diversity was lower in Samples 1–2 for both 16S and ITS profiles, consistent with the possibility that drying/storage and/or other sample-specific factors reduce detectable richness and evenness. A plausible explanation is partial DNA degradation during drying and long-term storage, which may disproportionately affect the detection of low-abundance taxa. Despite this, community composition remained broadly comparable across sample types: Supplemental Tables S1 and S2 show substantial overlap of classified taxa across all samples, including several genera detected in all seven samples (16S: *Blastococcus*, *Nocardioides*, *Marmoricola*, *Pseudonocardia*, *Sphingomonas*, *Paracoccus*, TM7a; ITS: *Trichoderma*, *Fusarium*, *Botryotrichum*, *Exophiala*, *Alternaria*, *Cladosporium*, *Malassezia*), alongside sample-specific enrichments present in both heat-dried and fresh concrete. Notably, many of these recurrent genera have been linked in the literature to concrete-relevant processes.

Concrete deterioration by acid attack primarily involves acidolysis, in which hydration products in the matrix react with infiltrating organic acids, leading to ion release and loss of solid mass [21]. This is followed by complexolysis, where the resulting acid ions form complexes with metal ions released during acidolysis. This facilitates further dissolution of the solid phase, while the precipitation of expansive reaction products may cause cracking. Among the tested acids, citric, succinic, and acetic acid have been reported as the most aggressive agents of concrete degradation [22]. However, the stability of original cement components and the extent of their recovery post-acid exposure remain unclear. While many heterotrophic bacteria (e.g., *Vibrio*, Acidobacteria, *Bacillus*) are known to produce organic acids that can induce decalcification of cement hydration products, none were detected at significant levels in our samples. Autotrophic bacteria such as *Nitrosomonas*, *Nitrobacter* (nitrifiers), and sulphur-oxidizers like *Thiobacillus*, *Thiothrix*, *Thiomicrospira*, and *Beggiatoa* produce inorganic acids, which are typically more corrosive than their organic counterparts. Previous studies have identified *Thiobacillus*, *Acidothiobacillus*, and *Thiomonas* as dominant taxa in corroded concrete sewer pipes [7,23], yet these were also not present at notable levels in our dataset. In contrast, fungi capable of producing organic acids were well represented. We identified six fungal genera previously associated with concrete biodeterioration (*Exophiala*, *Trichoderma*, *Alternaria*, *Penicillium*, *Aspergillus*, and *Fusarium*) at high relative abundances. Notably, *Fusarium* species have been implicated in the deterioration of concrete bridges along the Nile River [21], highlighting their potential role in structural degradation.

Microbial taxa identified in this study have also been explored for their self-healing potential, particularly through biologically induced mineralization. Among bacteria, species within the genus *Bacillus*, notably *Bacillus sphaericus* and *Bacillus pasteurii*, have garnered significant attention. These bacteria can induce calcium carbonate precipitation via ureolysis, where the enzymatic hydrolysis of urea results in the production of ammonium and carbonate ions [24]. In the presence of calcium ions, this leads to the formation of calcite, which can seal microcracks in concrete [25,26]. Other metabolic pathways used for biomineralization include denitrification and sulphate reduction, both of which also result in alkaline conditions favorable to calcium carbonate precipitation [27,28]. Interestingly, some fungal taxa may offer similar capabilities: *Aspergillus nidulans*, *Trichoderma reesei*, *Neurospora crassa* and other filamentous fungi have demonstrated the ability to precipitate calcium carbonate through organic acid-mediated dissolution and reprecipitation cycles, as well as through CO_2_ production during respiration [29,30,31]. The resulting alkalinity in localized microenvironments can support mineral precipitation. Furthermore, the hyphal structure of fungi may facilitate crack infiltration, increasing their utility in healing deeper or more extensive damage compared to bacterial systems. However, empirical evidence for the use of fungi in concrete self-healing remains limited, with most studies conducted at laboratory scale [32]. These mechanisms are primarily relevant for serviceability-scale microcracks where water ingress can occur. Autogenous healing is generally limited to cracks < 0.2 mm, whereas microbe-enabled mineralization approaches have been reported to extend healing into the sub-millimeter range [33,34,35]. Microbial activity is most likely to contribute under intermittent wetting that provides water for transport and activation, access to oxygen, and sufficient Ca^2+^/carbonate availability in the alkaline matrix to support CaCO_3_ precipitation. From an engineering perspective, these microbe–concrete interactions are most likely to affect performance by altering permeability and ion transport within the near-surface zone and crack network. Acid-driven decalcification and persistent biofilms may increase porosity and accelerate ingress of aggressive agents, whereas biomineralization-driven CaCO_3_ precipitation may reduce permeability and slow such ingress under serviceability conditions, potentially supporting longer-term durability even when complete crack closure is not achieved. The potential contributions of dominant fungal and bacterial genera to concrete healing or deterioration, together with their proposed mechanisms, are outlined in Table 3.

Engineering such microbial systems for self-healing applications involves several considerations. For bacteria, researchers have developed encapsulation techniques (e.g., lightweight aggregates, hydrogel beads, or silica microcapsules) to preserve viability and trigger calcite formation upon crack exposure. For fungi, similar strategies are being explored, but their larger size and different environmental requirements pose engineering challenges. Advances in synthetic biology and metabolic engineering may eventually allow the optimization of metabolic fluxes for improved mineralization efficiency and environmental adaptability. The microbial communities detected in our study, particularly the diverse fungal phyla, present an untapped reservoir of metabolic capabilities with implications for both concrete degradation and healing. However, further functional characterization of these taxa is needed to understand their precise roles in concrete–microbe interactions. A key future direction is to distinguish microbial taxa based on their functional outputs rather than just taxonomic identity. For instance, strains that efficiently secrete strong organic acids may be desirable in applications focused on controlled concrete degradation, such as biorecycling of demolition waste. Conversely, strains capable of biomineralization and structural repair should be prioritized for applications in infrastructure maintenance and sustainability. This duality also highlights the need for application-specific microbial engineering. For degradation purposes, communities could be selected or engineered for high acid production, low pH tolerance, and enzymatic activity targeting the cement matrix. For healing, strains might be selected for high urease activity, resilience in alkaline environments, and efficient calcium carbonate precipitation. Moreover, the high proportion of unclassified sequences in our dataset underscores a gap in our current understanding of microbial biodiversity in built environments. There is a clear need for expanded reference databases and experimental work, including isolation, culturing, and genome sequencing, to uncover the full functional potential of these microbes.

A key limitation of this study is the modest sample size, which constrains statistical power and limits the extent to which our observations can be generalized across the diversity of real structural concrete. Urban concrete varies widely in mix design and material properties (e.g., cement type and content, aggregates, supplementary cementitious materials, admixtures, porosity, and age), and these factors can shape microbial colonization by altering pH, moisture retention, and nutrient availability. In addition, exposure histories differ substantially among structures, including wet–dry cycling, de-icing salts and other ions, air pollution, temperature fluctuations, and UV exposure, each of which may select for different microbial assemblages. Our dataset, therefore, represents a snapshot of urban concrete microbiomes rather than a comprehensive survey across concrete types and exposure regimes. Future studies should use larger, stratified cohorts spanning defined concrete classes and controlled exposure metadata, ideally coupled with standardized fresh sampling and functional assays, to link community shifts to specific material and environmental drivers. Importantly, the functional roles discussed are inferred from amplicon-based taxonomic assignments and published literature on related taxa and were not experimentally validated in this study.

## 5. Conclusions

This study demonstrates that urban concrete supports diverse bacterial and fungal communities that tolerate its extreme environmental conditions. Although elemental composition did not significantly explain community variation, taxonomic profiling revealed a set of recurrent concrete-associated taxa, providing a shortlist of organisms that may be leveraged as minimally engineered starting points for concrete biotechnology. Several detected taxa are linked in the literature to processes relevant to both concrete deterioration and healing. Acid-producing and sulphate-oxidizing microorganisms may contribute to deterioration, while biomineralizing bacteria and filamentous fungi offer promising avenues for self-healing through calcium carbonate precipitation and crack infiltration. Concrete-associated microbiomes, therefore, represent an underexplored reservoir of functions with potential applications in sustainable construction. Future work should prioritize functional characterization through cultivation, genome-resolved analyses, and controlled assays to identify strains suitable for biodegradation or biogenic mineral formation. Improved taxonomic resolution, particularly for fungi, will further refine our understanding of these interactions. Harnessing microbial capabilities may ultimately support low-impact recycling strategies and the development of self-healing concrete materials, thereby reducing the environmental footprint of built infrastructure.

## Figures and Tables

**Table 1:** Description of the collected concrete samples.

Sample	Provenance	Location	Condition
Sample 1	Stadt Wien, MA 39	48°11′04.5″ N 16°24′22.5″ E	Heat-dried; Stored
Sample 2	Stadt Wien, MA 39	48°11′04.5″ N 16°24′22.5″ E	Heat-dried; Stored
Sample 3	Underground Parking Lot	48°12′00.7″ N 16°21′47.2″ E	Fresh
Sample 4	Demolition Site	48°13′18.2″ N 16°19′47.3″ E	Fresh
Sample 5	Demolition Site	48°13′04.0″ N 16°19′37.8″ E	Fresh
Sample 6	Demolition Site	48°12′02.8″ N 16°21′46.6″ E	Fresh
Sample 7	Demolition Site	48°12′01.0″ N 16°21′45.4″ E	Fresh

**Table 2:** 16S/ITS amplicon sequencing statistics.

Sample		Input Reads	Filtered Reads	Denoised Reads	Final Reads	Sequence Variants
Sample 1	16S	63,923	53,643	52,826	43,014	15,887
	ITS	1,325,494	1,052,753	1,049,949	980,093	777
Sample 2	16S	471,511	393,918	389,612	333,566	99,788
	ITS	1,335,182	995,567	986,967	838,949	3109
Sample 3	16S	678,977	456,705	450,260	414,685	88,365
	ITS	1,134,423	789,143	787,187	775,615	674
Sample 4	16S	650,845	494,005	475,252	419,182	148,961
	ITS	1,100,937	858,795	852,416	836,152	3608
Sample 5	16S	817,755	639,040	616,372	507,952	177,933
	ITS	549,658	425,822	418,815	384,765	2817
Sample 6	16S	450,299	331,680	314,895	268,269	106,315
	ITS	860,382	621,924	614,889	590,243	2953
Sample 7	16S	761,912	582,639	561,102	486,809	169,420
	ITS	999,341	721,341	712,288	693,586	1574

**Table 3:** Functional inference of the most abundant fungal and bacterial genera.

Kingdom	Phylum	Genera	Putative Microbial Activity	Mechanism of Concrete Healing/Degradation	Sources
Fungi	Ascomycota	*Exophiala*	Concrete degradation	Biofilm formation and secretion of acidic metabolites (organic acids) causing local scaling/decalcification of cementitious phases (including CaCO_3_ dissolution), potentially compounded by chelation-type metabolites; chemical weakening may enable hyphal in-growth that contributes to biomechanical degradation over time.	[36,37]
Fungi	Ascomycota	*Trichoderma*	Self-healing concrete	Spore-based activation upon crack formation (water/O_2_ ingress), followed by vegetative growth and CaCO_3_ biomineralization (calcite precipitation) on fungal cell walls/hyphae; hyphal networks provide extensive nucleation surfaces, promoting crack sealing.	[38,39]
Fungi	Ascomycota	*Fusarium*	Self-healing concrete, Concrete degradation	Dual role: (i) spore/growth-associated CaCO_3_ biomineralization (mineral deposition on hyphae/cell surfaces) promoting crack sealing; and/or (ii) secretion of organic acids and other metabolites that dissolve/decalcify cementitious phases, contributing to biodeterioration depending on environmental conditions and nutrient availability.	[38,40,41]
Fungi	Ascomycota	*Aspergillus*	Self-healing concrete, Concrete degradation	Dual role: (i) biomineralization via organic acid–mediated dissolution and reprecipitation cycles and/or CO_2_ production during respiration, resulting in CaCO_3_ deposition that can contribute to crack sealing; and (ii) biodeterioration via strong organic acid production leading to dissolution/decalcification of cement hydration products and local scaling.	[29,42]
Fungi	Ascomycota	*Penicillium*	Concrete degradation	Secretion of organic acids and associated metabolites that promote dissolution/decalcification of cementitious phases (acidolysis) and local scaling; may be enhanced under moist, nutrient-supplied conditions that support hyphal growth.	[43]
Fungi	Basidiomycota	*Malassezia*	No concrete-related activity reported	Not reported in concrete.	N/A
Fungi	Basidiomycota	*Armillaria*	No concrete-related activity reported	Not reported in concrete.	N/A
Bacteria	Actinobacteriota	*Blastococcus*	Self-healing concrete	Microcrack sealing via non-ureolytic MICP: respiration-driven alkalinity generation (bicarbonate production from organic carbon oxidation) promotes CaCO_3_ precipitation under high pH; precipitates may form as vaterite that transforms to stable calcite, reducing porosity/permeability and improving mortar strength while avoiding ammonia release associated with ureolysis.	[44]
Bacteria	Actinobacteriota	*Nitriliruptor*	Concrete degradation	Concrete-associated colonization/biofilm formation with metabolite-driven microenvironment changes (e.g., localized pH shifts) that can promote mineral dissolution/decalcification and microstructural weakening; mechanism likely indirect and context-dependent.	[45,46]
Bacteria	Firmicutes	*Veillonella*	No concrete-related activity reported	Not reported in concrete.	N/A
Bacteria	Actinobacteriota	*Arthrobacter*	Self-healing concrete	Crack remediation via CaCO_3_ biomineralization driven by amino-acid metabolism (oxidative deamination) and/or organic-acid utilization and ureolysis that elevates pH and carbonate ion availability; Ca^2+^ binds to negatively charged cell-wall constituents that serve as nucleation sites for crystal growth, reducing water permeability and sealing cracks.	[47,48]
Bacteria	Actinobacteriota	*Nocardioides*	Concrete degradation	Concrete colonization and biofilm-associated metabolic activity potentially contributing to biodeterioration via metabolite-mediated dissolution/decalcification of cementitious phases; effects are likely driven by localized microenvironment changes and are context-dependent.	[49,50]
Bacteria	Firmicutes	*Planomicrobium*	Self-healing concrete	Carbonatogenic activity observed (CaCO_3_ precipitation), consistent with bacteriogenic biomineralization; genus-specific evidence in concrete remains limited.	[51]

## Data Availability

The data presented in this study are openly available in [NCBI] at [https://www.ncbi.nlm.nih.gov/bioproject/PRJNA1288356/] (accessed on 2 January 2026), reference number [PRJNA1288356].

## Supplementary Materials

The following supporting information can be downloaded at: https://www.mdpi.com/article/10.3390/microorganisms14010131/s1, Figure S1: Physicochemical properties of concrete samples (extended); Figure S2: Correlation matrix of elemental concentrations in concrete samples; Figure S3: Fitted environmental vectors on the NMDS ordination of microbial communities; Table S1: Detailed 16S Community Composition; Table S2: Detailed ITS Community Composition; Table S3: Detailed Concrete Composition Analysis; Table S4: Detailed NMDS Statistics.

## Abbreviations

The following abbreviations are used in this manuscript:

ASV	Amplicon sequence variant
ITS	Internal transcribed spacer
LOD	Limit of detection
MICP	Microbially induced calcium carbonate precipitation
NMDS	Non-metric multidimensional scaling
rRNA	Ribosomal RNA
XRF	X-Ray fluorescence
