# Supplementary material for: Microbial Community Profiling of Concrete
Source: Microorganisms. 2026 Jan 7;14(1):131. doi: 10.3390/microorganisms14010131 (PMC12844258; doi:10.3390/microorganisms14010131)
Supplement: Supplementary file 1 [file microorganisms-14-00131-s001.zip › microorganisms-4078581-supplementary.pdf]

Microbial Community Profiling of Concrete

Supplementary Information

Caroline Danner*[1], Julien Charest*[1], Carlijn Borghuis[1], Philipp Aschenbrenner[2], Jakob Lederer[1], Robert L. Mach[1], Astrid R. Mach-Aigner[1],

* These authors contributed equally

[1] Institute of Chemical, Environmental and Bioscience Engineering, TU Wien, Gumpendorfer Strasse 1a, 1060 Wien, Austria

[2] Institute of Water Quality and Resource Management, TU Wien, Karlsplatz 13, 1040 Wien, Austria

Email addresses:

CD: caroline.danner@tuwien.ac.at

JC: julien.charest@tuwien.ac.at

CB: carlijn.borghuis@gmail.com

PA: philipp.aschenbrenner@tuwien.ac.at

JL: jakob.lederer@tuwien.ac.at

RLM: robert.mach@tuwien.ac.at

ARMA: astrid.mach-aigner@tuwien.ac.at

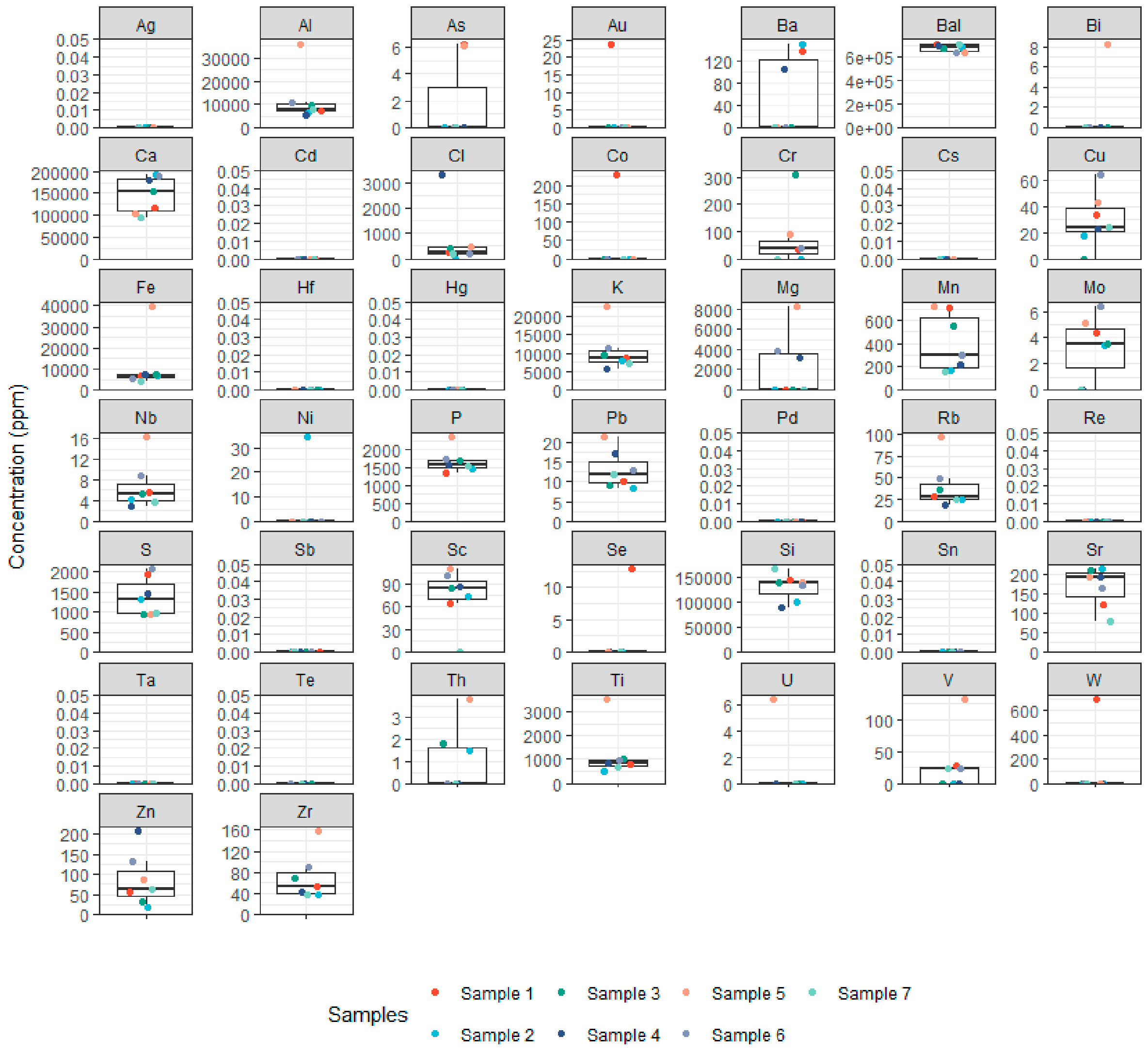

Supplementary Figure S1. Physicochemical properties of concrete samples (extended). Boxplots of elemental concentrations (ppm) for major and trace elements measured in concrete samples by XRF spectrometry, including Ag, Al, As, Au, Ba, Bal, Bi, Ca, Cd, Cl, Co, Cr, Cs, Cu, Fe, Hf, Hg, K, Mg, Mn, Mo, Nb, Nl, P, Pb, Pd, Rb, Re, S, Sb, Sc, Se, Si, Sn, Sr, Ta, Te, Th, Ti, U, V, W, Zn and Zr.

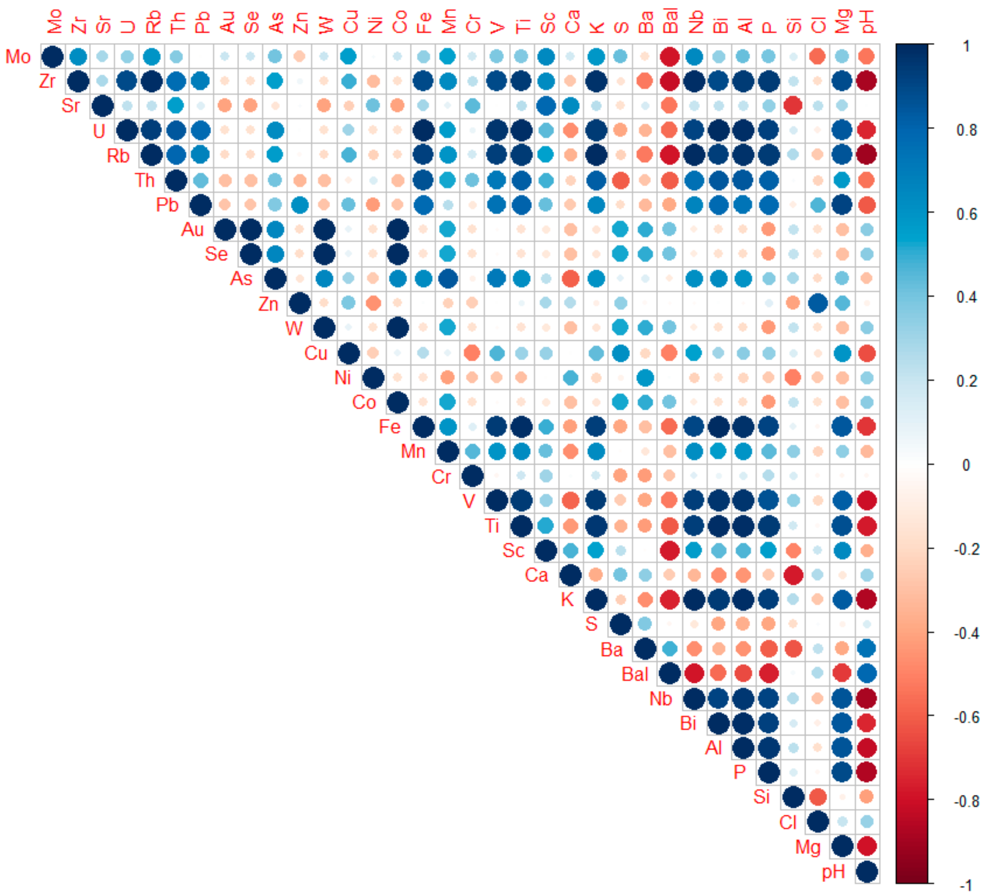

Supplementary Figure S2. Correlation matrix of elemental concentrations in concrete samples. Pairwise Pearson correlation coefficients among elemental concentrations (ppm) measured in concrete samples by XRF spectrometry. The upper triangular correlogram visualizes the strength and direction of correlations among pH and major and trace elements, including Al, As, Ba, Bal, Bi, Ca, Cl, Co, Cr, Cu, Fe, K, Mg, Mn, Mo, Nb, P, Pb, Rb, Re, S, Sc, Se, Si, Sr, Th, Ti, U, V, W, Zn and Zr.

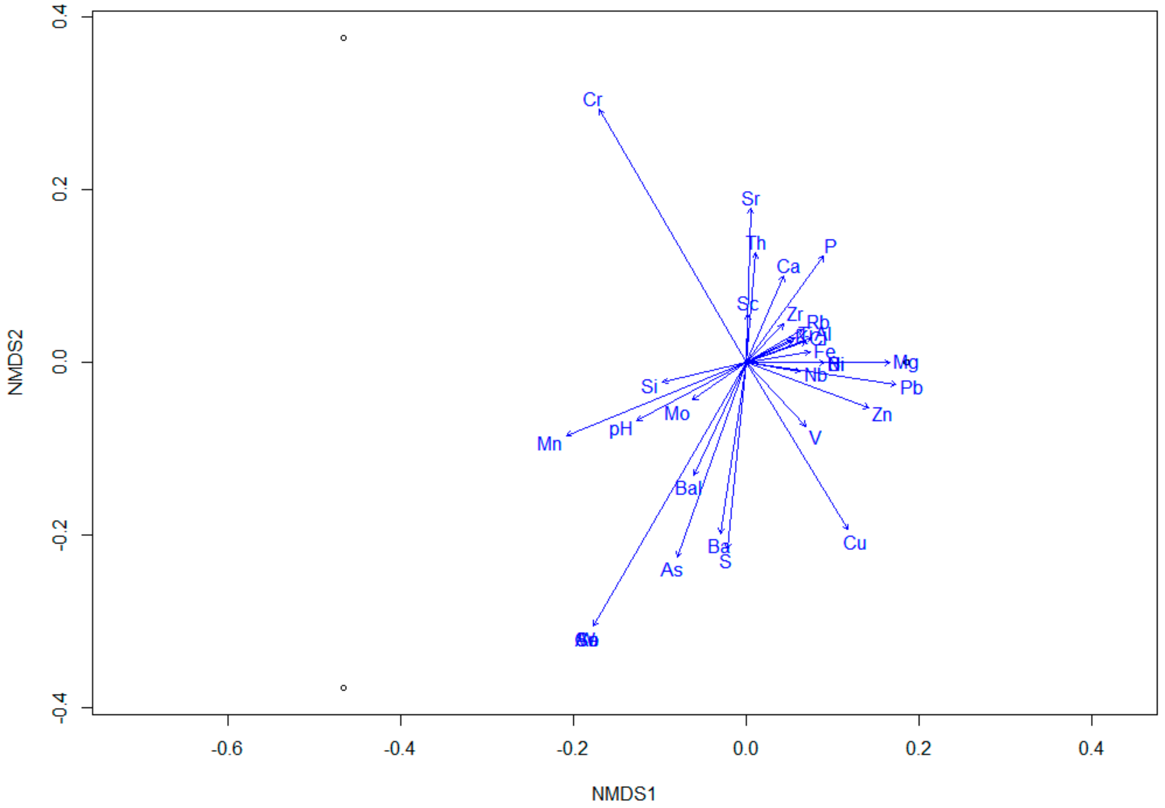

Supplementary Figure S3. Fitted environmental vectors on the NMDS ordination of microbial communities. Environmental variables were fitted onto the NMDS configuration using the envfit() function with all possible free permutations (n = 5,039). Vector orientation indicates the direction of maximum correlation between each physicochemical variable and community structure, while vector length and r^2 values represent the strength of these associations. No environmental variables showed significant correlations with NMDS axes under the full permutation test (all $p > 0.1$). Numerical values for NMDS coordinates, r^2, and permutation-derived p-values for each variable are provided in Supplementary Table 4.
